# Tanshinone I alleviates steroid-induced osteonecrosis of femoral heads and promotes angiogenesis: in vivo and in vitro studies

**DOI:** 10.1186/s13018-023-03934-y

**Published:** 2023-06-30

**Authors:** Kai Sun, Yuman Xue, Xin Zhang, Xiaodong Li, Jun Zhao, Xilin Xu, Xiaofeng Zhang, Fubiao Yang

**Affiliations:** 1 The First Department of Orthopedics and Traumatology, First Affiliated Hospital, Heilongjiang University of Chinese Medicine, Harbin, Heilongjiang China; 2 The Second Department of Rehabilitation, The Second Affiliated Hospital of Heilongjiang University of Chinese Medicine, Harbin, Heilongjiang China; 3 Graduate School, Heilongjiang University of Chinese Medicine, Harbin, Heilongjiang China; 4 The Third Department of Orthopedics and Traumatology, The Second Affiliated Hospital of Heilongjiang University of Chinese Medicine, Harbin, Heilongjiang China; 5 President’s Office, The Third Affiliated Hospital, Heilongjiang University of Chinese Medicine, No. 2, Xiangjiang Road, Harbin, Heilongjiang China; 6 Teaching and Research Section of Orthopedics and Traumatology, Heilongjiang University of Chinese Medicine, No. 24, Heping Road, Harbin, Heilongjiang China

**Keywords:** Steroid, Osteonecrosis of the femoral head, Tanshinone I, Angiogenesis, SRY-box transcription factor 11

## Abstract

**Background:**

The impaired blood supply to the bones is an important pathological feature of steroid-induced osteonecrosis of the femoral head (SIONFH). Danshen is a Chinese herb that shows therapeutic effects on SIONFH, but the effects of one of its major bioactive constituents, Tanshinone I (TsI), on SIONFH remain unknown. Here, we evaluated the effects of TsI on SIONFH, particularly focusing on its effects on angiogenesis, in in vivo and in vitro research.

**Methods:**

SIONFH was induced in Sprague–Dawley rats by an intramuscular injection of methylprednisolone (40 mg/kg) in combination with an intraperitoneal injection of lipopolysaccharide (20 μg/kg). Morphological alterations of the femoral head were observed by dual-energy X-ray absorptiometry and HE staining. Western blot, qRT-PCR, and immunohistochemical/immunofluorescence staining were used to determine gene expression.

**Results:**

TsI (10 mg/kg) alleviated bone loss and rescued the expression of angiogenesis-related molecules (CD31, VWF, VEGF, and VEGFR2) in the femoral heads of SIONFH rats. Notably, TsI rescued the down-regulated expression of SRY-box transcription factor 11 (SOX11) in CD31^+^ endothelial cells in the femoral heads of SIONFH rats. In vitro studies showed that TsI preserved the dexamethasone-harmed angiogenic property (migration and tube formation) of human umbilical vein cells (EA.hy926), suppressed dexamethasone-induced cell apoptosis, reduced pro-apoptotic proteins (cytosolic cytochrome C, Bax, and caspase 3/9) and increased anti-apoptotic protein Bcl-2, whereas silencing of SOX11 reversed these beneficial effects.

**Conclusions:**

This study demonstrates that TsI alleviates SIONFH and promotes angiogenesis by regulating SOX11 expression. Our work would provide new evidence for the application of TsI to treat SIONFH.

**Graphical Abstract:**

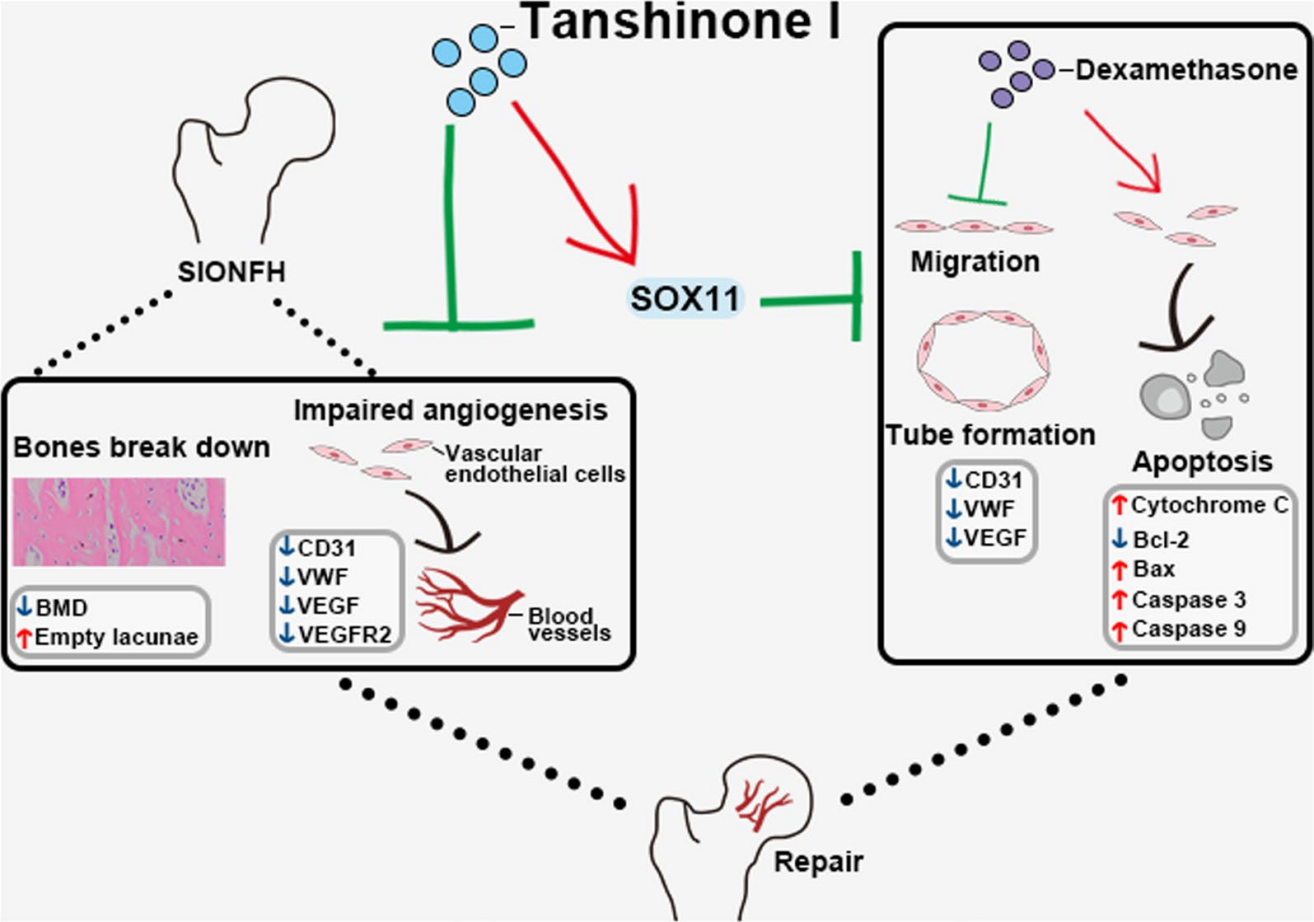

**Supplementary Information:**

The online version contains supplementary material available at 10.1186/s13018-023-03934-y.

## Background

Osteonecrosis of the femoral head (ONFH) is a debilitating skeletal disorder that commonly occurs in young and middle-aged individuals [1, 2]. Although many advances have been made in joint-preserving treatments, many patients still require surgery, usually total hip arthroplasty, while the durability of commonly used bone graft materials is unsatisfactory [3–9]. Steroids are known to be the most common cause of non-traumatic ONFH [10–12]. According to a multicenter investigation, among patients diagnosed with non-traumatic ONFH, 26.35% of males and 55.75% of females have reported corticosteroid use, and glucocorticoid intake is positively associated with an increased risk of non-traumatic ONFH [13]. Although several hypotheses have been proposed, the etiology and pathology of steroid-induced osteonecrosis of the femoral head (SIONFH) are not completely clarified [14].

The impaired blood supply to the bones is considered to be the main cause of ONFH [15]. To date, a variety of studies have revealed the correlation between steroids and deficient blood supply to bones. Steroids can exert direct toxicity on the microvasculature, induce endothelial cell apoptosis and lead to the dysfunction of vascular endothelial cells [16–20]. It has been reported previously that the growth, migration, in vitro tube formation capacity, and cytokine secretion of circulating endothelial progenitor cells are impaired in patients with SIONFH [21]. In addition to the direct effect on endothelial cells, steroids regulate some essential factors such as vascular endothelial growth factor (VEGF) to influence angiogenesis [22–24]. In contrast, promoting angiogenesis has been proven to significantly relieve SIONFH [25–27]. Therefore, enhancing angiogenesis is a promising approach for the prevention or early treatment of SIONFH.

Tanshinone I (TsI) is an important lipophilic diterpene extracted from Danshen (*Radix Salvia miltiorrhiza*). Danshen is an essential component of the Chinese herbal Huogu formula, which has beneficial effects in the treatment of SIONFH [28]. Danshen has also been found to promote angiogenesis in a rabbit model of the avascular necrotic femoral head [29]. Additionally, TsI has been reported to inhibit osteoclast differentiation and reduce the formation of multinuclear osteoclasts [30, 31]. Consistently, Danshen ethanolic extract, in which TsI has been identified as one of the major components, has been reported to reduce the lipopolysaccharide (LPS)-induced dental alveolar bone resorption in rats [32]. These findings indicate the potential beneficial effect of TsI in preventing osteonecrosis. Nonetheless, the specific role of TsI in angiogenesis in OFNH has not been mentioned yet. Therefore, investigating the effects of TsI on vascular endothelial cells and angiogenesis in ONFH is of significance.

SRY-box transcription factor 11 (SOX11) participates in embryonic development and promotes nerve regeneration [33, 34]. Moreover, it promotes tumor angiogenesis through transcriptional regulation of platelet-derived growth factor A in mantle cell lymphoma [35–37]. It has also been reported that SOX11 overexpression enhances the osteogenesis of tendon-derived stem cells and stimulates the tube formation capacity of HUVEC cells [38]. Additionally, an obvious decrease in SOX11 expression has been observed in dexamethasone (DEX)-treated bone marrow mesenchymal stem cells [39]. These findings suggest that SOX11 might be a positive regulator of angiogenesis, which supports the therapeutic potential of SOX11 in SIONFH. Interestingly, TsI has been found to dramatically reverse interleukin (IL)-1β-induced down-regulation of SOX11 in chondrocytes, thereby inhibiting chondrocyte inflammation and apoptosis and preventing the development of arthritis [40]. However, whether TsI has the same effect on SOX11 has not been investigated in SIONFH.

In the present study, we aimed to explore the effects of TsI on angiogenesis in SIONFH and to determine whether SOX11 is involved in the functions of TsI, hoping that our findings will be helpful for the prevention and early treatment of SIONFH.

## Methods

### Animal grouping and treatment

The protocol of animal experiments was approved by the Ethics Committee of the Heilongjiang University of Chinese Medicine and performed according to the guidelines for the care and use of experimental animals. Twelve-week-old male Sprague–Dawley rats (weighing 420 ± 20 g) were purchased from Liaoning Changsheng biotechnology co., Ltd. (SPF grade) and kept in cages under standard laboratory conditions (the temperature at 24℃, 12-h day/night cycle). Rats had free access to food and water. After one week of adaptive feeding, these rats were randomly divided into the control, SIONFH, and SIONFH + TsI groups (12 rats in each group). SIONFH models were established as previously described, and rats were intraperitoneally injected with lipopolysaccharide (LPS; 20 μg/kg/d) for two consecutive days and subsequently received an intramuscular injection of methylprednisolone (MPS; 40 mg/kg/d, Pfizer bio, China) for three consecutive days [41]. Rats in the control group were administered an equivalent amount of 0.9% saline at the same time.

Four weeks after the last injection of MPS, rats in the SIONFH + TsI group were intraperitoneally injected with 10 mg/kg TsI (CAS: 568-73-0, purity ≥ 98%, Aladdin, China) dissolved in 1% dimethyl sulfoxide (DMSO) once a day for four consecutive weeks. Rats in the control group and SIONFH group were administered an equivalent amount of vehicle (1% DMSO) at the same time. After the last injection of MPS, the body weight of the rats was measured at weeks 0, 1, 2, 3, 4, 5, 6, 7, and 8. Four consecutive weeks after the TsI injection, all rats were killed and the femoral heads were collected for later examination.

### Morphology evaluation

Dual-energy X-ray absorptiometry was used to determine the bone mineral density (BMD) of the femoral heads. Hematoxylin–eosin (HE) staining was performed for histological analysis. Briefly, bone tissue samples were fixed in 10% formalin, decalcified in ethylene diamine tetra-acetic acid (EDTA; Sigma, USA), embedded in paraffin, and sectioned at 4 μm. To observe the pathological changes in the bone tissues, the stained sections were examined under a light microscope (Olympus, Japan), and the rate of empty lacunae was calculated as previously described [42]. Ten fields of each section were randomly selected (under 200 × magnification), and twenty bone lacunae were counted in each field. The rate of empty lacunae was calculated as the number of empty bone lacunae versus the total number of bone lacunae.

### Immunohistochemical staining

Immunohistochemical staining was performed to detect angiogenesis-related proteins in the femoral head, including platelet endothelial cell adhesion molecule-1 (also known as CD31) and von Willebrand factor (VWF). The sections were incubated with primary antibodies against CD31 (1:100-diluted, A0378, ABclonal, China) and VWF (1:100-diluted, AF3000, Affinity, China) at 4 °C overnight, followed by incubation with a secondary antibody, horseradish peroxidase-conjugated goat-anti-rabbit IgG (1:500-diluted, #31460, ThermoFisher, USA) for 60 min. The sections were then incubated with the peroxidase substrate diaminobenzidine (Solarbio, China) for 5 min and counterstained with hematoxylin (Solarbio, China). Finally, the brown-yellow reaction products were observed under a light microscope (Olympus, Japan) at 400 × magnification.

### Immunofluorescence staining

Sections were blocked in goat serum for 15 min at room temperature. The sections were then incubated with primary antibodies against SOX11 (1:100-diluted, DF8614, Affinity, China), CD31 (1:100-diluted, 66,065-2-Ig, Proteintech, China), VWF (1:50-diluted, sc-365712, Santa Cruz, USA) and RUNX family transcription factor 2 (RUNX2; 1:50-diluted, 20,700-1-AP, Proteintech) at 4 °C overnight. Subsequently, the sections were incubated with Alexa Fluor™ 555-labeled goat-anti-rabbit secondary antibody (1:200-diluted, A27039, Invitrogen, USA) and fluorescein isothiocyanate (FITC)-labeled goat-anti-mouse secondary antibody (1:200-diluted, ab6785, Abcam, UK) at room temperature for 60 min. Finally, the samples were counterstained with 4', 6-diamidino-2-phenylindole (Aladdin, China) and photographed with a fluorescence microscope (Olympus, Japan) at 400 × magnification.

### Cell culture and treatment

The human umbilical vein cell line EA.hy926 is one of the most commonly used human vascular endothelial cell lines [43]. EA.hy926 was maintained in Dulbecco's modified Eagle's medium (Sigma, USA) supplemented with 10% (*v*/*v*) fetal bovine serum (Sigma, USA) at 37 °C under a 5% CO_2_ atmosphere. According to a previous study, treatment with 10 μM DEX for 48 h significantly induced cell apoptosis and impaired the angiogenic properties of EA.hy926 cells [25]. To determine the effects of TsI on cells exposed to DEX (Aladdin, China), we incubated the cells with 20 μM TsI and/or 10 μM DEX for 48 h. To determine the role of SOX11 in angiogenesis, cells were transfected with SOX11-specific siRNA (si-SOX11) or negative control siRNA (si-NC) using Lipofectamine™ RNAiMAX Reagent (Invitrogen, USA) according to the manufacturer’s instructions.

### Cell counting kit-8 (CCK-8) assay

Cell viability was measured by the CCK-8 assay. EA.hy926 cells were seeded into 96-well plates and incubated with 0, 1, 5, 10, 20, 40, and 80 μM TsI for 48 h. Subsequently, 10 μl CCK-8 reagent (Beyotime, China) was added to the culture medium in each well. One hour later, A450 was analyzed using a microplate reader (BIOTEK, USA) to represent the cell viability.

### EA.hy926 migration assay

The migration of EA.hy926 cells was evaluated by the wound-healing assay. Cells were cultured in the serum-free medium until they reached 100% confluence, and a straight scratch was made across the middle of each well using a 200-μl pipette tip. The cells were then cultured in the serum-free medium containing 20 μM TsI and/or 10 μM DEX for 24 h. At the time points of 0 and 24 h, images of cells at identical locations were acquired under a phase-contrast microscope (Olympus, Japan) at 100 × magnification, and the migration rate was calculated.

### EA.hy926 tube formation assay

EA.hy926 cells were seeded into 96-well plates precoated with Matrigel (Corning, USA) at a density of 1 × 10^4^ cells per well. Cells were then cultured in the serum-free medium containing 20 μM TsI and/or 10 μM DEX for 16 h. Finally, images of capillary-like structures were obtained under a phase-contrast microscope (Olympus, Japan) at 100 × magnification, and the number of tubes was calculated.

### Cell apoptosis assay

The Annexin V-FITC/propidium iodide (PI) kit (KeyGEN, China) was used to evaluate cell apoptosis according to the manufacturer's instructions. After 48 h of incubation with 20 μM TsI and/or 10 μM DEX, the cells were harvested, washed with PBS, and resuspended in the binding buffer. Next, the cells were incubated with Annexin V-FITC/PI for 5–15 min in the dark. Finally, the cells were analyzed individually using a flow cytometer (ACEA, USA).

### RNA extraction and quantitative real-time PCR (qRT-PCR)

Total RNA was extracted from bone tissues or transfected EA.hy926 cells using an RNApure high-purity total RNA rapid extraction kit (BioTeke, China) following the manufacturer’s protocol. Afterward, total RNA was reverse-transcribed into cDNA using M-MLV reverse transcriptase and RNase inhibitor (Takara, Japan). qRT-PCR was conducted using Taq HS Perfect Mix (Takara, Japan) and SYBR Green (BioTeke, China) in an Exicycler 96 PCR system (BIONEER, Korea). Relative quantification of gene expression was determined using the 2^−ΔΔCt^ method. β-actin was employed as a housekeeping gene for internal normalization. Primer sequences (5′–3′) are as follows: Rat *Sox11*, forward-AGGATGCCGACGACCTCATG, reverse-GAAGTTCGCCTCCAGCCAGT; Human *SOX11*, forward-ACGGTCAAGTGCGTGTTTCTG, reverse-TGCTGGTGCGGTGGTTCCTC; Human CD31 (gene name *PECAM1*), forward-AAGATAGCCTCAAAGTCG, reverse-CTGGGCATCATAAGAAAT.

### Western blot

Bone tissues or EA.hy926 cells were lysed in RIPA lysis buffer supplemented with PMSF (Beyotime, China). The cytosolic fraction was prepared using a Subcellular Structure Mitochondrial Extraction Kit (BOSTER, China) according to the manufacturer’s instructions. Protein samples were then quantified using a BCA protein assay kit (Beyotime, China). Proteins were loaded on SDS-PAGE and transferred to PVDF membranes (ThermoFisher, USA). The membranes were then blocked with 5% bovine serum albumin (BSA; Biosharp, China) and incubated with primary antibodies against VEGF (1:1000-diluted, A16703, ABclonal, China), vascular endothelial growth factor receptor 2 (VEGFR2; 1:1000-diluted, A5609, ABclonal), Cytochrome C (1:1000-diluted, A4912, ABclonal), Bax (1:1000-diluted, A19684, ABclonal), Bcl-2 (1:500-diluted, A0208, ABclonal), Caspase 3 (1:500-diluted, AF7022, Affinity), Caspase 9 (1:1000-diluted, #9505, Cell Signaling Technology, USA), CD31 (1:1000-diluted, A0378, ABclonal), VWF (1:1000-diluted, AF3000, Affinity), SOX11 (1:1000-diluted, A17945, ABclonal), and β-actin (1:2000-diluted, 60008–1-Ig, Proteintech) at 4 °C overnight. Finally, the membranes were incubated with secondary antibodies at 37 °C for 40 min, and proteins were visualized using an enhanced chemiluminescence kit (7 sea Biotech, China).

### Statistical analysis

Data are presented as mean ± standard deviation (SD). Statistical analysis was performed using GraphPad Prism. One-way analysis of variance (ANOVA) followed by Tukey's post hoc test was used to compare differences between groups. Values with *P* < 0.05 were considered statistically significant.

## Results

### TsI alleviated bone loss in SIONFH rats

**Fig. 1:**
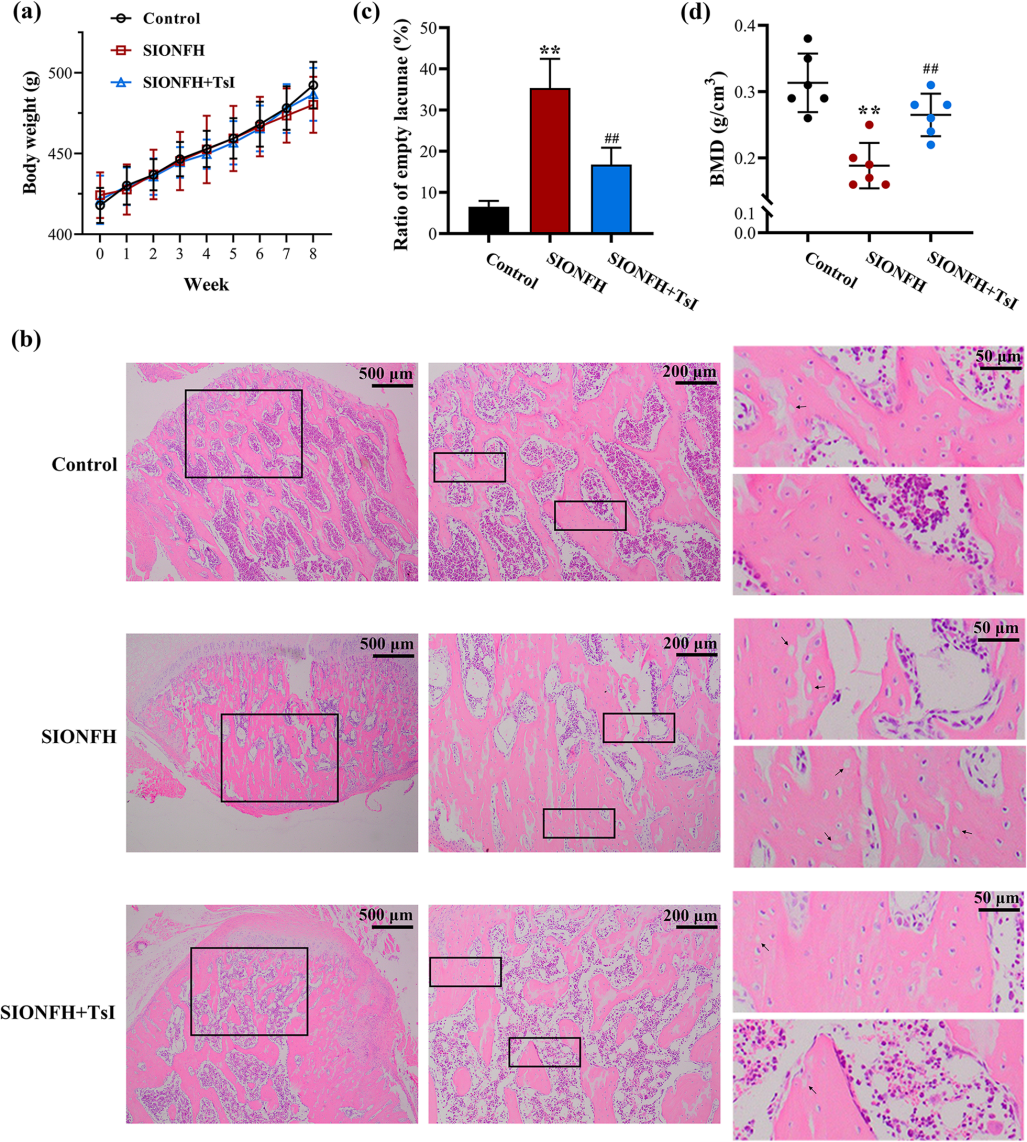
TsI alleviated bone loss in SIONFH rats. **a** Body weight of rats in each group. **b** HE staining showed the histological appearance of femoral heads (magnification 40 ×, scale bar: 500 μm; magnification 100 ×, scale bar: 200 μm; magnification 400 ×, scale bar: 50 μm). The empty lacunae are indicated by black arrows. **c** The rate of empty lacunae was calculated. **d** Femoral head bone mineral density (BMD) was measured by dual-energy X-ray absorptiometry. **P < 0.01 *vs.* Control and ^##^*P* < 0.01 *vs.* SIONFH. *N* = 6 in each group

Body weight changes during the study period were shown in Fig. 1a. No significant difference was observed among the groups. According to the results of HE staining, fewer empty lacunae were observed in the SIONFH + TsI group compared with the SIONFH group (Fig. 1b and c). Dual-energy X-ray absorptiometry results showed that BMD in the SIONFH group was lower than that in the control group, whereas TsI treatment increased the BMD of SIONFH rats (Fig. 1d). These results indicated that TsI treatment alleviated bone loss in rats with SIONFH.

### TsI promoted angiogenesis in the femoral heads of SIONFH rats

**Fig. 2:**
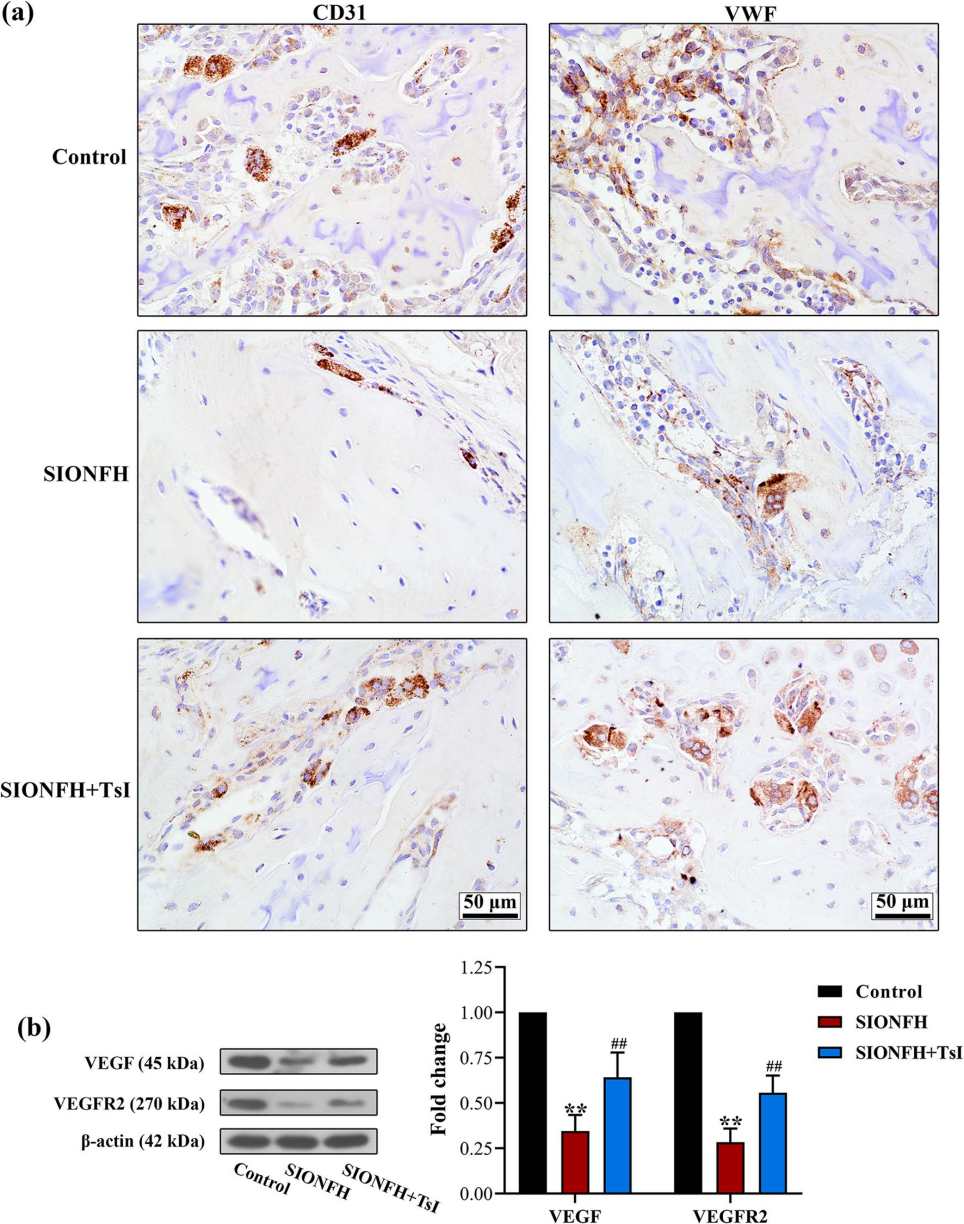
Effects of TsI on angiogenesis in femoral heads of rats with SIONFH. **a** Immunohistochemical staining for CD31 and VWF in femoral heads of rats (magnification 400 ×; scale bars: 50 μm). **b** Western blot analysis for expression levels of VEGF and VEGFR2 in femoral heads of rats. **P < 0.01 *vs.* Control and ^##^*P* < 0.01 *vs.* SIONFH. *N* = 6 in each group

We subsequently investigated the effects of TsI on angiogenesis in the femoral heads by evaluating the expression of angiogenesis-related molecules. Immunohistochemical staining showed an obvious decrease in CD31 and VWF in the femoral heads of rats with SIONFH, whereas TsI treatment significantly attenuated the loss of CD31 and VWF (Fig. 2a). Moreover, TsI markedly rescued the MPS-induced reduction in VEGF and VEGFR2 in the femoral heads (Fig. 2b). These results suggested that TsI promoted angiogenesis in the femoral heads of rats with SIONFH.

We further performed immunofluorescence double staining for the osteogenic marker RUNX2 and angiogenesis-related molecules. As shown in Additional file 1: Fig. S1, both CD31 (Additional file 1: Fig. S1a) and VWF (Additional file 1: Fig. S1b) co-localized with RUNX2. The expression of CD31, VWF, and RUNX2 in the SIONFH group was lower than that in the control group, whereas TsI treatment rescued the expression of CD31, VWF, and RUNX2. These results indicated that TsI restored the reduction of CD31 and VWF not only in vascular endothelial cells but also in osteoblasts.

### TsI increased the SOX11 expression in the femoral heads of rats with SIONFH

**Fig. 3:**
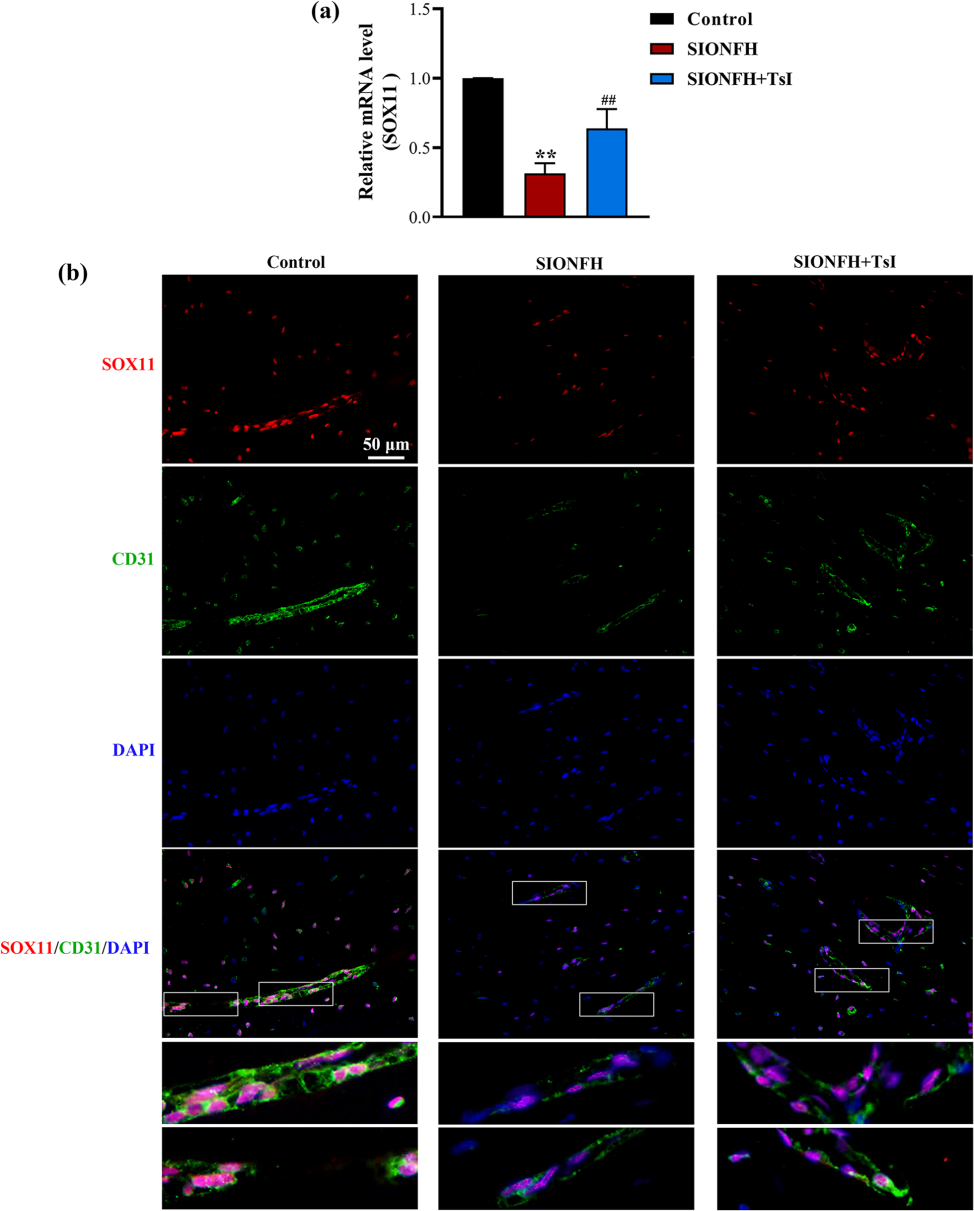
Effects of TsI on SOX11 expression in femoral heads of rats with SIONFH. **a** The qRT-PCR was used to measure the mRNA levels of *Sox11* in the femoral heads of rats. **P < 0.01 *vs.* Control and ^##^*P* < 0.01 *vs.* SIONFH. *N* = 6 in each group. **b** Immunofluorescence staining for SOX11 and CD31 (an endothelial cell marker) in femoral heads of rats (magnification 400 ×; scale bar: 50 μm)

Next, we examined the effects of TsI on SOX11 expression in the femoral heads of SIONFH rats. As shown in Fig. 3a, the mRNA level of *Sox11* was significantly reduced in the femoral heads of rats with SIONFH and was markedly increased by TsI treatment. Immunofluorescence staining showed that SOX11 expression significantly decreased in CD31-positive endothelial cells, whereas TsI treatment elevated SOX11 expression (Fig. 3b). These results suggested that SOX11 might be involved in the effects of TsI on angiogenesis in the femoral head of SIONFH rats.

### TsI suppressed the apoptosis in DEX-treated EA.hy926 cells

**Fig. 4:**
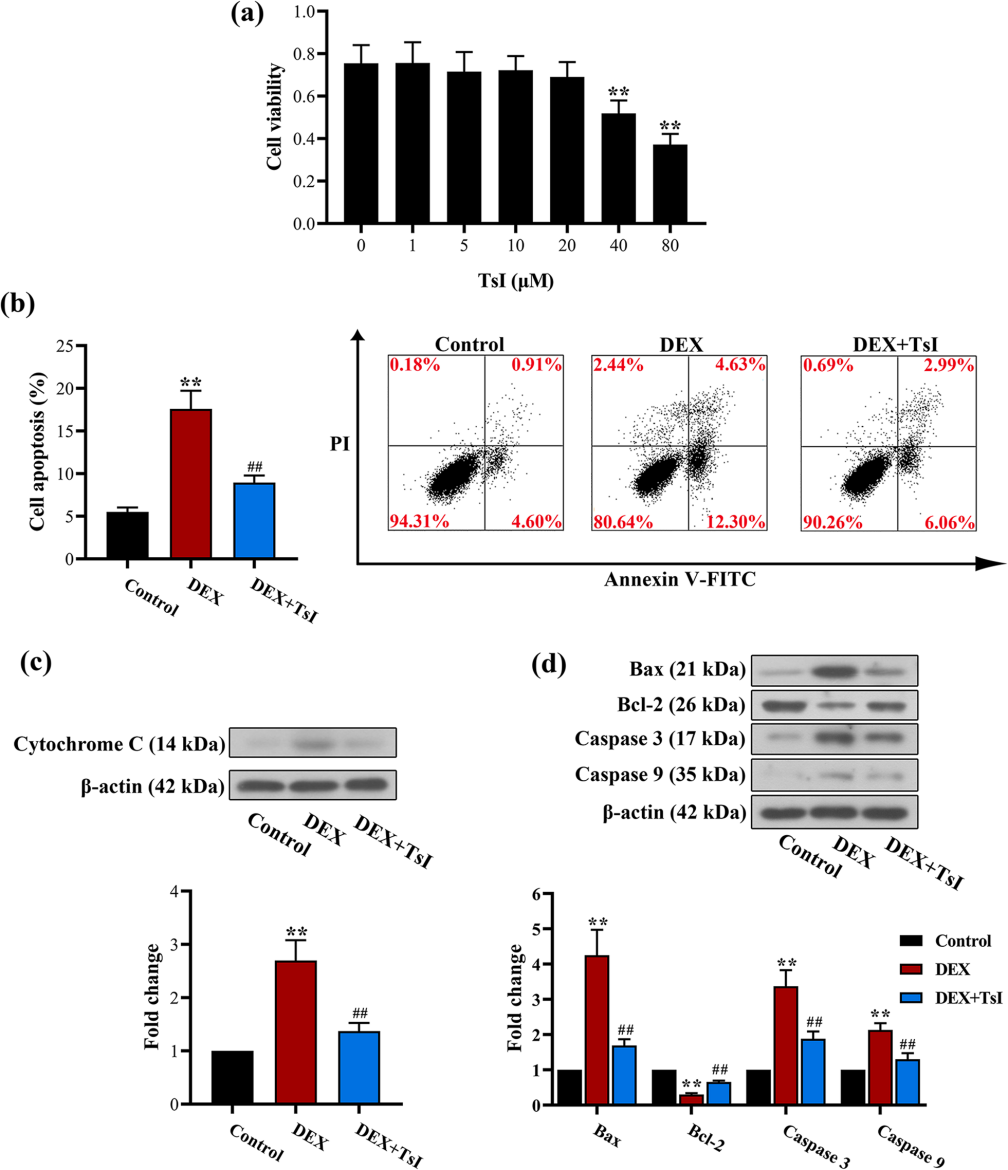
Effects of TsI on DEX-induced apoptosis of EA.hy926 cells. **a** Cell viability was detected by the CCK-8 assay after incubation with different concentrations (0, 1, 5, 10, 20, 40, 80 μM) of TsI for 48 h. **P < 0.01 *vs.* 0 μM TsI. EA.hy926 cells were treated with 20 μM TsI and/or 10 μM DEX for 48 h. **b** The apoptosis rate was measured by Annexin V-FITC/PI flow cytometry. **c**, **d** Western blot analysis was performed to evaluate the expression levels of cytosolic cytochrome C, Bax, Bcl-2, caspase-3, and caspase-9 in EA.hy926 cells. **P < 0.01 *vs.* Control and ^##^*P* < 0.01 *vs.* DEX. *N* = 3 in each group

Considering the critical role of endothelial cells in angiogenesis [44], we used EA.hy926 cells to mimic the ONFH endothelial condition in vitro and investigated the effects of TsI on EA.hy926 cells exposed to DEX. The CCK-8 assay indicated that less than 20 μM TsI did not show obvious cytotoxicity on EA.hy926 cells (Fig. 4a). The apoptosis rate was significantly increased in cells exposed to DEX, whereas 20 μM TsI markedly decreased the apoptosis rate (Fig. 4b). Western blot analysis revealed that TsI significantly decreased the levels of pro-apoptotic proteins (cytosolic Cytochrome C, Bax, Caspase 3, and Caspase 9) and rescued the expression of anti-apoptotic protein Bcl-2 in DEX-treated EA.hy926 cells (Fig. 4c, d). These findings suggested that TsI markedly protected EA.hy926 cells from DEX-induced apoptosis.

### TsI rescued the angiogenic property of DEX-treated EA.hy926 cells

**Fig. 5:**
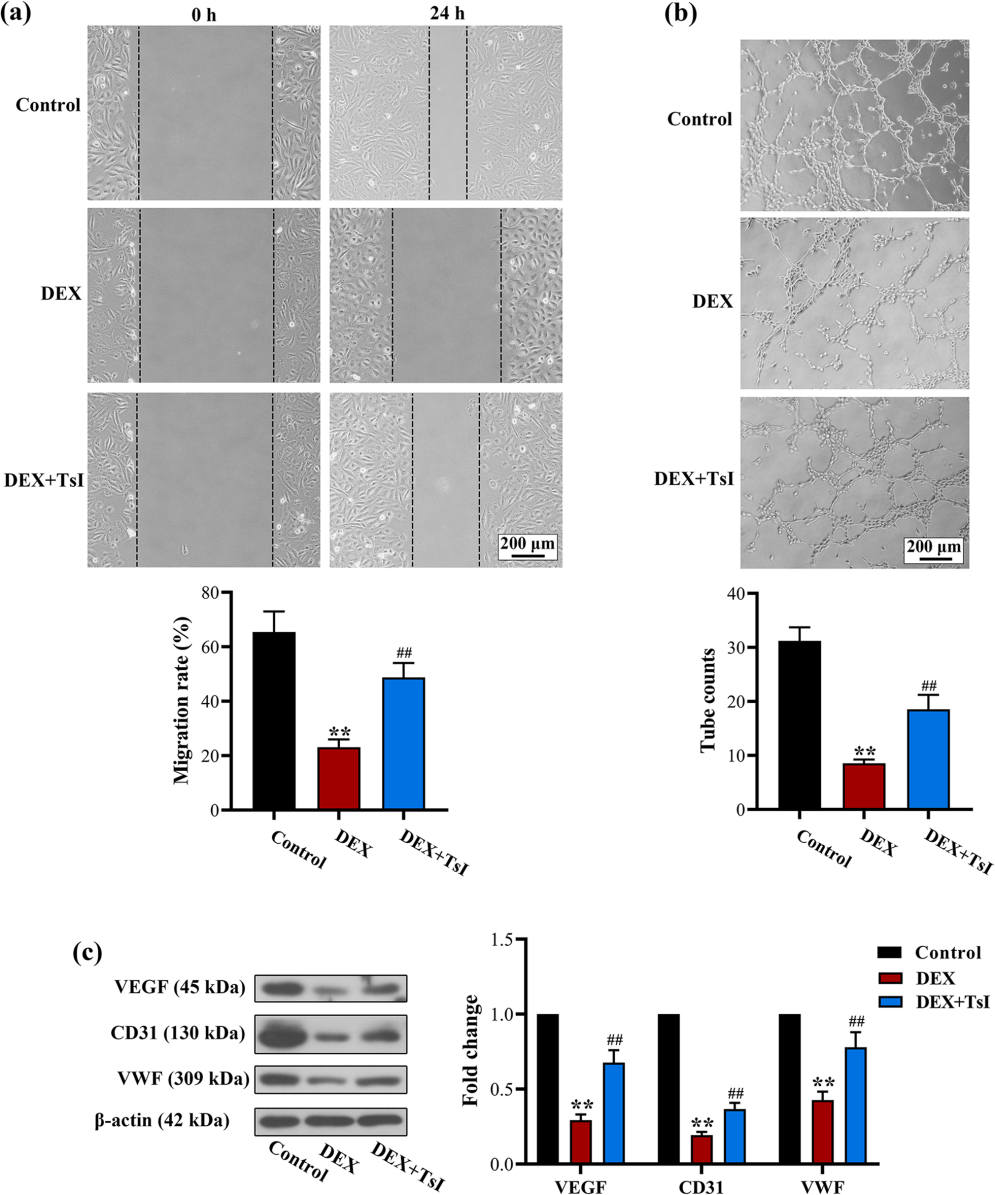
Effects of TsI on the angiogenic property of EA.hy926 cells exposed to DEX. EA.hy926 cells were treated with 20 μM TsI and/or 10 μM DEX for 48 h. **a** The wound-healing assay showed the migration capability of EA.hy926 cells. **b** The tube formation assay was used to measure the angiogenesis activity of EA.hy926 cells. **c** The expression levels of VEGF, CD31, and VWF were measured by Western blot analysis. Magnification 100 ×; scale bars: 200 μm. **P < 0.01 *vs.* Control and ^##^*P* < 0.01 *vs.* DEX. *N* = 3 in each group

Next, wound-healing and tube formation assays were applied to evaluate the effects of TsI on the angiogenesis activity of EA.hy926 cells. As shown in Fig. 5a, the DEX-damaged migration capacity of EA.hy926 cells was rescued by TsI treatment. In the tube formation assay, an obvious decrease in loop formation was observed in cells exposed to DEX in comparison to control cells, whereas more visible tubes were observed in cells receiving TsI protection, indicating the promoting effect of TsI on the angiogenic property of EA.hy926 cells (Fig. 5b). Consistently, the protein levels of VEGF and VEGFR2 were reduced by DEX, whereas TsI treatment preserved their expression (Fig. 5c). These findings indicated that TsI rescued the angiogenic property of DEX-treated EA.hy926 cells.

### Down-regulation of SOX11 reversed the protective effects of TsI on DEX-treated EA.hy926 cells

**Fig. 6:**
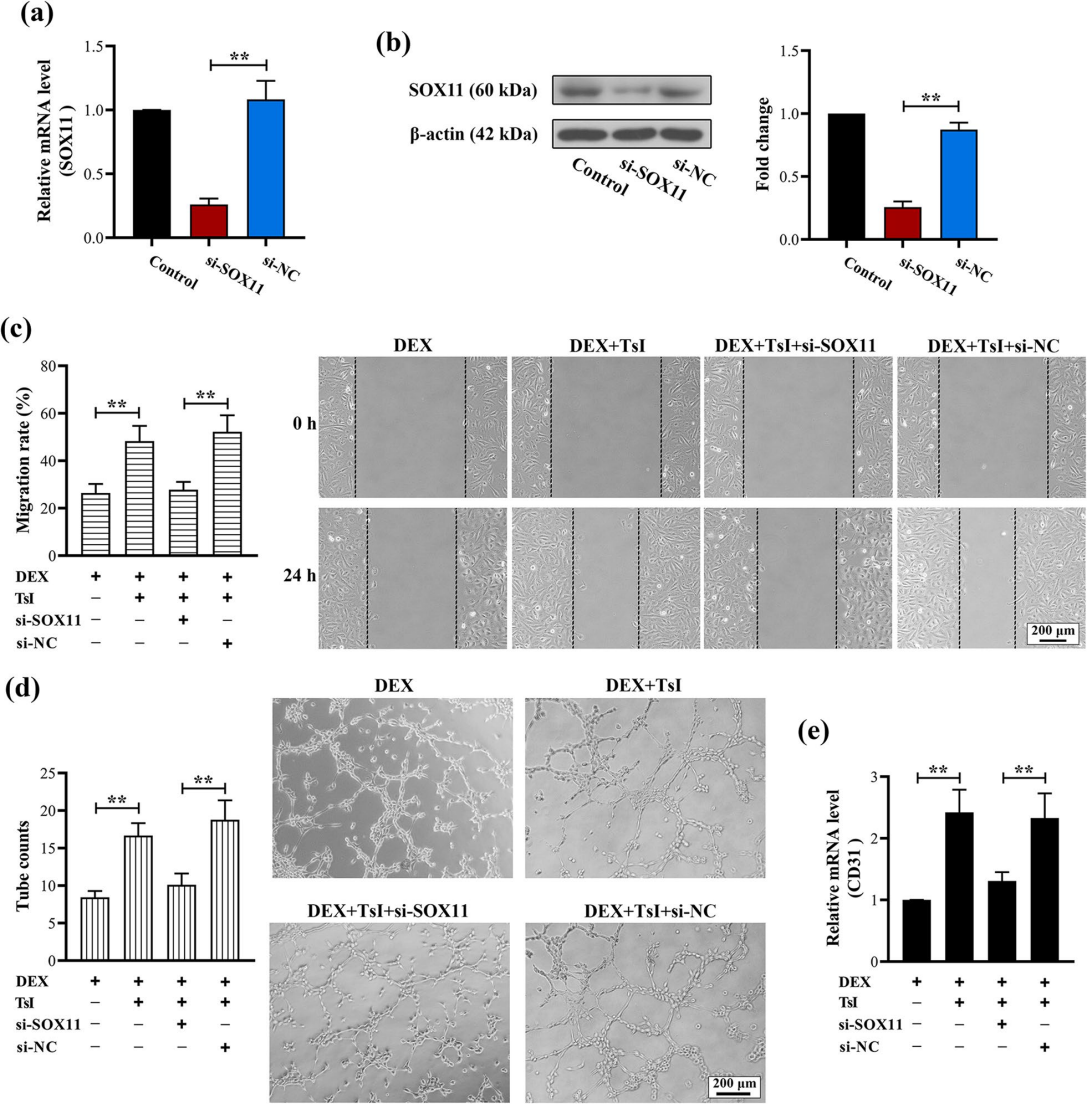
Down-regulation of SOX11 impaired the protective effects of TsI on EA.hy926 cells exposed to DEX. EA.hy926 cells were transfected with siRNA against SOX11 or its negative control. Forty-eight hours after cell transfection, **a**, **b** qRT-PCR and Western blot were used to determine the expression of *SOX11* in EA.hy926 cells. **c**, **d** Wound-healing and tube formation assays were performed to determine the migration capability and angiogenesis activity of EA.hy926 cells, respectively. **e** The mRNA level of CD31 was detected by qRT-PCR. Magnification 100 ×; scale bars: 200 μm. **P < 0.01. *N* = 3 in each group

To determine whether SOX11 is involved in the protective effects of TsI on DEX-treated EA.hy926 cells, siRNA against SOX11 or its negative control was transfected into EA.hy926 cells. Forty-eight hours after transfection, qRT-PCR and Western blot analysis confirmed the down-regulation of SOX11 (Fig. 6a and b). Knockdown of SOX11 impaired TsI-induced preservation of migration (Fig. 6c), tube formation (Fig. 6d), as well as CD31 expression (Fig. 6e) in DEX-treated EA.hy926 cells. These results indicated that SOX11 was important for the protective effects of TsI on the angiogenic property of EA.hy926 cells exposed to DEX.

## Discussion

Long-term steroid medication is the leading cause of non-traumatic ONFH, but its pathogenesis remains unclear [13]. In the present study, we determined the role of TsI, a major bioactive component of Danshen (a Chinese herbal medicine), in promoting angiogenesis in in vivo and in vitro models of SIONFH. In our work, MPS in combination with LPS was used to establish a rat model of SIONFH, as the efficacy of this method has been proved in the previous research [41]. We found that TsI administration significantly improved the histopathological characteristics of ONFH in rats, including empty lacunae and bone marrow cell necrosis. In addition, TsI treatment dramatically increased the BMD of rats with SIONFH. These results strongly suggested that TsI ameliorated steroid-induced bone loss of the femoral head in rats.

Steroid-induced impairment of angiogenesis is one of the main causes of ONFH [15]. Angiogenic factors play essential roles in angiogenesis. VEGF and its receptor VEGFR2 can convey signals that promote the proliferation, survival, and migration of endothelial cells, thereby accelerating the formation of new blood vessels during bone repair [45]. Other endothelial markers such as CD31 and VWF also promoted angiogenesis [46]. In the serum samples of patients with SIONFH, these angiogenesis-related factors were decreased [47], thereby disrupting vascularization and new blood vessel growth into the necrotic bone [48].

As previously reported, injection of the VEGFR2 antibody into the capsular attachment to the proximal femur successfully induced a rat model of ONFH [49]. In contrast, treatment to promote the expression of these angiogenic proteins was confirmed to enhance angiogenesis and prevent the progression of ONFH [50–52]. Interestingly, Danshen was found to promote angiogenesis by increasing the expression of VEGF in a rabbit model of the avascular necrotic femoral head [29]. Consistently, we found that TsI treatment significantly reversed the steroid-mediated inhibition of the expression of these angiogenesis-related factors in in vivo and in vitro models of SIONFH, suggesting that TsI may play a protective role in SIONFH by promoting angiogenesis.

Suppression of endothelial cell apoptosis is required for the maintenance of blood vessel integrity and angiogenesis [44], whereas steroids significantly induced the apoptosis of endothelial cells [25, 53]. Mechanistically, DEX increased the expression levels of apoptosis indicators (Bax and cytosolic Cytochrome C) in endothelial cells [54, 55]. In contrast, factors that inhibit apoptosis were shown to protect the angiogenic properties of endothelial cells [53].

TsI treatment was found to suppress the pro-apoptotic effects of paraquat in SH-SY5Y cells and to prevent paraquat-induced alterations in Bax, Bcl-2, and cytosolic Cytochrome C [56]. Similarly, in the present work, we found that TsI treatment rescued endothelial cells from DEX-induced apoptosis and markedly decreased pro-apoptotic proteins and increased anti-apoptotic proteins. Moreover, TsI treatment significantly reversed the DEX-mediated inhibition of endothelial cell migration and tube formation, indicating the strong promotion of the angiogenic property of endothelial cells. Therefore, we speculated that the protective role of TsI in SIONFH was partially mediated through the inhibition of endothelial cell apoptosis.

To date, the molecular mechanisms underlying the pharmacological effects of TsI have not been fully elucidated. Some studies have reported the potential receptors for TsI, for example, TsI was found to directly target and exhibit inhibitory activities against insulin-like growth factor 1 receptor in vascular smooth muscle cells and epidermal growth factor receptor/fibroblast growth factor receptor 4 in HEK293 cells, but these interactions have not been verified in vascular endothelial cells [57, 58]. In this work, we investigated the regulation of SOX11 expression by TsI.

SOX11 is a SOXC transcription factor that plays an important role in skeletogenesis and neurogenesis [59, 60]. Recent evidence has implicated SOX11 in angiogenesis [38]. In SOX11-positive mantle cell lymphoma, increased tumor angiogenesis and higher levels of pro-angiogenic factors, including angiopoietin-1 and -2 and fibroblast growth factor-1, were observed. Additionally, SOX11 could transcriptionally up-regulate the expression of platelet-derived growth factor A, thereby promoting vessel formation in endothelial cells [35, 36, 61]. Furthermore, SOX11 was identified as a negative regulator of cell apoptosis, as it inhibited the activity of caspases, including caspase 3, 6, and 7, whereas knockdown of SOX11 significantly induced cell apoptosis [62–65].

In a model of IL-1β-induced murine osteoarthritis, SOX11 was significantly down-regulated by IL-1β in chondrocytes, whereas TsI treatment reversed this down-regulation and protected chondrocytes from IL-1β-induced apoptosis [40]. Similarly, we found that silencing of SOX11 impaired the TsI-mediated protection of the angiogenic property of endothelial cells, suggesting an essential role of SOX11 in the protective effects of TsI.

However, other molecules or signaling pathways may also be implicated in TsI-mediated protective roles in angiogenesis. For instance, TsI inhibited the phosphorylation of NF-κB, and activation of NF-κB caused endothelial cell death and apoptosis and led to the inhibition of angiogenesis [66–68]. Therefore, the beneficial functions of TsI in angiogenesis in SIONFH may also be related to the inhibition of NF-κB signaling, which requires further research. This study has some limitations. Considering that hyperactivation of osteoclasts is intensively implicated in the progression of SIONFH [69, 70], it is necessary to investigate whether the therapeutic effect of TsI on SIONFH is related to its functions of inhibiting osteoclast differentiation [30, 31].

## Conclusions

This study reported the beneficial effects of TsI in treating SIONFH. In vivo studies showed that TsI attenuated bone loss and promoted angiogenesis in the femoral heads of SIONFH rats. In vitro studies showed that TsI protected endothelial cells from steroid-induced apoptosis and preserved the angiogenic properties of the cells. Mechanistic studies demonstrated that SOX11 was implicated in these protective effects of TsI.

## Supplementary Information

**Additional file 1**: **Fig. S1**. Effects of TsI on angiogenesis-related molecules in osteoblasts in femoral heads of rats with SIONFH. Immunofluorescence double staining for the osteogenic transcription factor RUNX2 and **a** CD31 and **b** VWF in femoral heads of rats (magnification 400×; scale bars: 50 μm).

## Data Availability

All data generated or analyzed during this study are included in this published article.

## Abbreviations

ONFH: Osteonecrosis of the femoral head
SIONFH: Steroid-induced osteonecrosis of the femoral head
VEGF: Vascular endothelial growth factor
TsI: Tanshinone I
SOX11: SRY-box transcription factor 11
IL: Interleukin
LPS: Lipopolysaccharide
MPS: Methylprednisolone
DMSO: Dimethyl sulfoxide
BMD: Bone mineral density
HE: Hematoxylin-eosin
EDTA: Ethylene diamine tetra-acetic acid
FITC: Fluorescein isothiocyanate
VWF: Von Willebrand factor
DEX: Dexamethasone
CCK-8: Cell counting kit-8
PI: Propidium iodide
qRT-PCR: RNA extraction and quantitative real-time PCR
VEGFR2: Vascular endothelial growth factor receptor 2
SD: Standard deviation
ANOVA: One-way analysis of variance
NF-κB: Nuclear factor κB
RUNX2: RUNX family transcription factor 2
