# Supplementary figures and images for: Tanshinone I alleviates steroid-induced osteonecrosis of femoral heads and promotes angiogenesis: in vivo and in vitro studies
Source: J Orthop Surg Res. 2023 Jun 30;18:474. doi: 10.1186/s13018-023-03934-y (PMC10311706; doi:10.1186/s13018-023-03934-y)

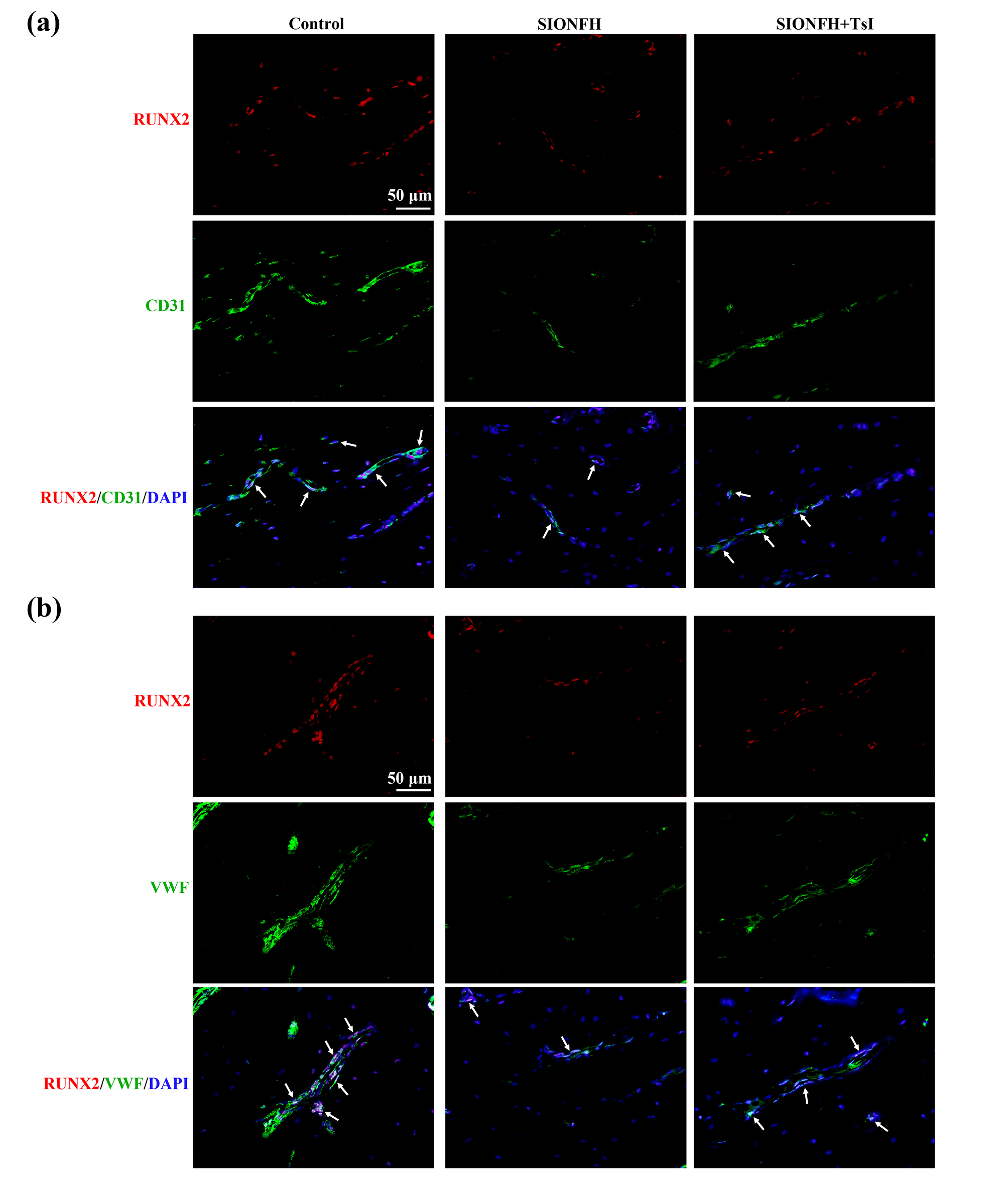

Supplement: Supplementary file 1 — Additional file 1: Fig. S1. Effects of TsI on angiogenesis-related molecules in osteoblasts in femoral heads of rats with SIONFH. Immunofluorescence double staining for the osteogenic transcription factor RUNX2 and a CD31 and b VWF in femoral heads of rats (magnification 400×; scale bars: 50 μm). [file 13018_2023_3934_MOESM1_ESM.tif]
